# Medicine, Religion, and the Humanitarian Ethos: Walter B. Cannon, Unitarianism, and the Care of Spanish Republican Refugees in France

**DOI:** 10.1093/jhmas/jrac002

**Published:** 2022-04-21

**Authors:** Jon Arrizabalaga, Àlvar Martínez-Vidal

**Affiliations:** Milá y Fontanals Institution for Research in Humanities (IMF-CSIC), Barcelona, Spain; López Piñero Inter-university Institute, University of Valencia, Valencia, Spain

**Keywords:** Humanitarian Ethos, Medicine and Religion, Medical Aid, Unitarianism, Walter B. Cannon, Spanish Civil War and Republican Exile in France

## Abstract

Biographies of Walter B. Cannon (1871-1945) usually present two sides of his life: one, where he was an outstanding man of science in the United States during the so-called “Golden Age of Medicine,” and the other, where he was a leading humanitarian activist engaged in myriad causes, notably in the defense of Spanish democracy during the Civil War (1936-1939). However, these biographies fail to take into account that the apparent link between these two sides of his life was his religious conviction.

This study summarizes the aims and accomplishments of the American Medical Bureau to Aid the Spanish Democracy (AMBASD) of which Cannon was chair between 1937 and 1939. Then, it examines Cannon’s inspirational role on the international relief work with Spanish Republican refugees in France, through the case of the Varsovie Hospital of Toulouse that between 1945 and 1949 was jointly managed by the Unitarian Service Committee (USC) and the Joint Anti-Fascist Refugee Committee (JAFRC), and renamed Varsovie Hospital/Walter B. Cannon Memorial in recognition of the Spanish Republicans’ debt for his extraordinary contribution during the Spanish Civil War and beyond. Finally, the article investigates the Unitarian roots of Cannon’s humanitarian ethos by exploring the historical relations of this religious movement with science and with many major actors at Harvard University as well as the links of Cannon's relatives to Unitarianism.

The analysis reveals Unitarianism’s influence on Cannon’s views about science, democracy, and liberty, as well as on his remarkable involvement in the medical solidarity movement with the Second Spanish Republic and other similar commitments. In sum, it shows how important is to branch out in our studies of medical and scientific practice to include practitioners’ broader social and religious communities in order to understand their motivations, achievements, and behavior.

Walter B. Cannon (1871-1945), the prestigious Harvard Medical School professor who devoted most of his long and fruitful academic life to research and the popularization of his theories on human physiology, has usually been presented as having two distinct sides: he was an outstanding man of science in the United States during the so-called “Golden Age of Medicine,”^1^ and a leading humanitarian activist in the defense of Spanish democracy during the Civil War (1936-1939) and other causes abroad. Surprisingly enough, the link between these two sides of Cannon’s life appears to be his religious conviction.

The analysis in this article can be distilled into four important and previously unexamined points. Firstly, Cannon’s Unitarian faith was inseparable from and indeed considerably influenced his tenure as a scientific worker, a socio-political activist, and a medical humanitarian volunteer. Secondly, the tragedy of the Spanish Civil War made him, like many other scientists around the world, turn toward non-communist leftism in defending democracy from fascist totalitarianism. Thirdly, Cannon concerned himself with the fate of Spanish Republican exiles, especially his physiologist colleagues, until his death in October 1945, just after the end of the Second World War. Finally, but significantly, his memory continued to inspire the efforts to provide humanitarian relief to the Spanish Republican exiles in France through adding the mention of Cannon Memorial to the name of the Varsovie Hospital until the 1950, when the geopolitical logic of Cold War ended up prevailing over any other consideration.

A summary of the aims and accomplishments of the American Medical Bureau to Aid the Spanish Democracy (AMBASD) that Cannon chaired between 1937 and 1939 comes first. This loose coalition of liberal, socialist, and communist relief associations had been founded in 1936, formed on the basis of medical solidarity in the United States with Spanish Republicans.^2^

Secondly, an examination is given of Cannon’s inspirational role on the international relief work with Spanish Republican refugees in France, which was jointly implemented by the Unitarian Service Committee (USC) and the Joint Anti-Fascist Refugee Committee (JAFRC) through the case of the Varsovie Hospital of Toulouse. The USC was founded in Boston in 1940 as part of the American Unitarian Association to assist European refugees fleeing from Nazi persecution. The JAFRC was formed in 1941 by Lincoln Battalion veterans of the Spanish Civil War to provide aid to Spanish Loyalists refugees from Francoist Spain.^3^ In October 1944, the French Forces of the Interior (FFI) and the exiled Spanish *guerrilleros* outfitted an old chateau in a working class quarter of Toulouse as a hospital to look after Spanish wounded and sick fighters and, eventually, Spanish civilian refugees in need of healthcare. It became popularly known as the *Hôpital Varsovie*, based on its location on *rue Varsovie*. In 1946, soon after Cannon’s death, the *Hôpital Varsovie* was renamed Varsovie Hospital/Walter B. Cannon Memorial, in recognition of the Spanish Republicans’ debt for his extraordinary contribution during the Spanish Civil War and beyond.^4^ The reference to the eminent US scientist was removed without explanation from the new name of the Varsovie Hospital at the end of 1949, one year and a half after the USC had chosen, in February 1948, to withdraw from the project. By 1950, despite Cannon’s previous popularity in Toulouse, the rigors of the Cold War had vanished in France his memory as a prototype of an antifascist American scientist committed to humanitarian aid for the Spanish republican cause and as a firm supporter of understanding between peoples all over the world. Then, very few people in France – and probably in the United States, too – might have known that Unitarian faith was a major inspiration on Cannon’s life journey. And this oblivion has endured to this day. In fact, historians so far have missed the religious elements of Cannon's humanitarian and scientific ethos, so that their understandings of his deep, lifelong political commitment to various humanitarian causes are incomplete.

Finally, the study investigates the Unitarian roots of Cannon’s humanitarian ethos by exploring the historical relations of this Christian theological movement with science and with actors at Harvard University as well as the links of Cannon's relatives to Unitarianism. Altogether, the analysis reveals Unitarianism’s influence on his views about science, democracy, and liberty as well as on his remarkable involvement in the medical solidarity movement with the Second Spanish Republic and other similar commitments.

## International Medical Aid to the Spanish Republicans from the United States

It is well known that the Spanish Civil War served as a testing ground for new war machines and combat strategies later to be used in WWII. Germany and Italy systematically tested their armaments on the battlefield as well as against the civilian population in the republican rear-guard. It was additionally a testing ground for medical and other technologies for the protection of civilians in the context of bombings of towns and cities, mass evacuations, and rising famine. For instance, indirect blood transfusions by means of storable bottles, without the need for the donor to be alongside the wounded soldier in the field hospital, were first performed by actors on both sides during the Spanish Civil War.^5^ This innovation was later put into practice on a large scale for the first time in the United Kingdom by the physiologist Janet Vaughan (1899-1993), who was aided by Frederic Duran Jordà (1905-1957), the former director of the Barcelona Blood-Transfusion Service. This took place at the Hammersmith Hospital in London, on the occasion of the London blitz bombings by the German *Luftwaffe* in 1940 and 1941.^6^

Located in the rear-guard, Spanish Republican cities and villages were increasingly bombed by the German air force and the Italian navy. They aimed to simultaneously demoralize the troops loyal to the Republic and test the efficacy of their war machines. Hundreds of localities were attacked on gunnery exercises that principally targeted municipal markets, fuel tanks, railway stations and, of course, working-class neighborhoods. The most well-known case of these gunnery exercises was that of the Basque town of Guernica, literally devastated by the German Condor Legion. The lesser-known repeated bombings that left thousands of victims in their wake in big cities such as Madrid, Barcelona, and Valencia, and smaller ones such as Alicante or Almeria, must not be forgotten either. Passive defense of civilian populations led to the building of countless underground refuges in the Republican towns to protect people from the bombings and the resulting blasts, shrapnel, and fires, as well as from an eventual chemical warfare attack.^7^

The news disseminated by international press agencies, as well as photographic and cinematographic reports, which showed the suffering of the Spanish civilian population in the rear of both contending sides, quickly aroused numerous humanitarian aid initiatives throughout the world, including those in the United States, France, Great Britain, Ireland, Norway, Sweden, and Switzerland. During this time, Western democracies selectively practiced non-intervention policies, leaving the work to independent aid agencies. Many of these agencies (such as the Red Cross International Committee, Save the Children Fund and Save the Children International Union, American Friends Service Committee, and the Mennonite Relief and Service Committee) rushed to send staff and material resources (food, medicine, clothing) to help the most vulnerable, especially women and children, all through the war. These aid agencies mostly operated on the Republican side, not least because the Francoist authorities chose to channel foreign relief through *Auxilio Social*, a governmental organization inspired on the Nazi model. It should be added that neutrality was the main characteristic of the aid provided by the American Friends, so their humanitarian efforts were deployed on both sides.^8^

The actions of the AMBASD – chaired by Cannon^9^– could be seen as a Spanish Civil War antecedent to the actions of the Unitarian Service Committee in its support of refugees in France during WWII and its aftermath in the case of refugees fleeing the Spanish Republic. The role of the Spanish Civil War in relation to WWII draws a parallel to the role of the AMBASD in relation to the USC. Emerging in the United States, and inspired by the ideal of health internationalism, both institutions aimed to respond to the needs of civilians – women, children, disabled veterans, the elderly, the sick – afflicted by the circumstances of war and ignored by the international politics of the time.

## Chairing the AMBASD During the Spanish Civil War

In 1936, after the military rebellion led by Franco – with the support of Hitler and Mussolini – against the democratically elected Spanish government, a multifaceted crisis arose throughout Spain, chiefly affecting Republican areas. There was growing doubt in the US of the ability of the American Red Cross, and the International Committee of the Red Cross (ICRC), to provide an effective response to the challenges of the medical, health, food, and humanitarian crises triggered by the Spanish Civil War. The policies of non-intervention of France and Great Britain, which the US supported through its Congress’s Neutrality Act, *de facto* favored the insurgents. As a perverse consequence of the Non-Intervention Agreement signed by major world powers in August 1936, the rebels could rely, from the very start, on the support of Germany and Italy, while the Spanish Republic could not buy or stock up on arms. By being the single power willing to sell those weapons, the USSR managed to increase its political influence on the successive Republican governments in the course of the war, both on the battlefront and in the rearguard. Cannon was very critical of the British and French for their lack of intervention and of the strict international neutrality of the Americans. He argued that they had “refused to the legitimate Spanish government the right, recognized in international law, to purchase arms for its own defense.”^10^ This meant that the only way for the American people to help the Spanish Republic was to arrange for humanitarian relief. This consisted mostly of personnel and sanitary equipment sent to the camps by individuals or groups unaffiliated with the American government. Cannon’s autobiographical testimony placed the AMBASD right in the middle of the difficult situation:

In the circumstances the only action friends of the Spanish Republic could take was that of providing medical and surgical supplies, clothing, and food. For more than two years I served as national chairman of the Medical Bureau to Aid Spanish Democracy. During that time we sent to Spain medical personnel and surgical instruments, hospital equipment and ambulances, amounting in value to more than a million dollars.^11^

The AMBASD’s aim was, in Cannon’s words, to “[provide] medical and surgical supplies, clothing and food” to the Loyalist government of the Spanish Republic “in its struggle for liberty” against Franco’s military rebellion and “his ruthless fascist collaborators.” The coalition brought one hundred individuals –physicians, surgeons, dentists, nurses, ambulance drivers, and other healthcare personnel to Spain.^12^ It was led by Edward Barsky (1897-1975),^13^ a surgeon at the Beth Israel Hospital in New York City, and Fredericka Martin (1905-1992), a chief nurse and administrator. The AMBASD provided medical care to the almost three thousand men of the Lincoln Brigade, in addition to supporting Republican Spain by shipping drugs, ambulances, and medical equipment from the United States to palliate the sufferings of the war victims.^14^

Cannon chaired the AMBASD beginning in March 1937, and then co-chaired the Medical Bureau and North American Committee to Aid Spanish Democracy. The latter became the leading US relief coalition to help Spain, after the New York-based AMBASD and the North American Committee to Aid Spanish Democracy (NACASD, also founded in 1936) merged to form this wider committee early in 1938. It acted as an umbrella for a variety of groups including ethnic organizations and labor unions, which contributed funds, medical supplies, food, and ambulances to Spain. Until his resignation from his double leadership position in mid-April 1939, Cannon appeared to have been the most prominent US physician publicly supporting Spanish Loyalists.^15^ He was kept closely informed about what was going on in Spain, mostly through his close friendships with two Spanish colleagues, Juan Negrín (1892-1956)^16^ and Jesús M. Bellido (1880-1952).^17^ The former was the premier of the Republic during the war years (beginning in May 1937), and the latter was a professor of physiology at the University of Barcelona and the secretary of Religious Cults (*Comisario de Cultos*) of the Republican Government at the end of the Civil War.

Despite the fact that his commitment to the Spanish Democracy raised “much hostile criticism” in the US conservative medical establishment both inside and outside Harvard, Cannon did not waiver in his determination, and always insisted that his time and efforts devoted to this cause were well spent:

Naturally enough I was charged with being a Bolshevik, a supporter of communism, an enemy of the Roman Catholic church, and in general a Red, with all the dark insinuations that applied to that term. Because of the many letters which had to be written, the many conferences and committee meetings which had to be attended, and because of the gatherings which had to be addressed in Philadelphia, Detroit, and elsewhere, and also because of the political controversy, this whole experience was very time-consuming. I do not regret, however, having followed the course I have just outlined; I only regret that our efforts were not effective in helping at last to achieve the common degrees of freedom among a people long held in ignorance and economic repression.^18^

After the end of the Spanish Civil War, the AMBASD was dissolved, along with other transnational organizations that had supported the Republicans. However, the events that followed over the course of the next few years in Europe, particularly in France, where more than 150,000 Spanish refugees were stranded, led to new solidarity groups being set up in North America. More or less leftist, these groups aimed to support the refugees by means of awareness campaigns, fundraising, sending them food and medicine, and even rescuing those in greatest danger. It was under these circumstances that Edward Barsky founded the JAFRC in 1942, a relief organization grouping together former members and sympathizers of the Lincoln Brigade as well as supporters of the Republican exiles in France and Mexico.^19^

## Inspiring the Humanitarian Action of the USC in Post-War WWII

The creation of the USC in 1940 was a result of both the verified information that citizens from Nazi-occupied European countries were being persecuted for their political beliefs or solely for being Jews, and the prevailing apathy in US public opinion toward refugees in Europe.^20^ The USC was a member of the American Unitarian Association (hereafter AUA), whose founding executive director, Robert Dexter (1887-1955), was a North American social worker who had been building the AUA’s international and social engagement as of 1927, and who had been continuously involved in humanitarian efforts since WWI.^21^ The USC founding coincided with, and was indeed prompted by, the Nazi military invasion of northern and western France. The occupation of Paris led to the Armistice on 22 June 1940 and to the establishment of the Vichy Regime. It was at this point that thousands of foreign refugees, along with French citizens fleeing occupied areas, poured into unoccupied southern France, where they converged with the Spanish Republican exiles.

The USC established its first European office in Lisbon, with the Reverend Charles R. Joy (1885-1978) at its helm. The city was the capital of a neutral country, as well as the site of one of the few important non-military harbors in continental Europe that was still in operation. In early 1941, Joy appointed Noel Field (1904-1970) as director of a second office in Marseille where the USC’s relief mission for France would be set up. Throughout the course of WWII, Joy and Field, in collaboration with other humanitarian organizations and many refugee volunteers, developed healthcare, social relief, and emigration programs across southern France for refugees escaping from the Nazis and their allied regimes all over Europe.^22^ Its humanitarian work was initially focused on helping refugees escape arrest, torture, or even death, and reach safety. Through the war and early post-war years, its relief tasks increasingly pivoted toward medical aid, including sending in medical personnel, drugs, sanitary equipment, and setting up field hospitals.

It appears all began in February 1939, when AUA minister Waitstill Sharp (1902-1983) and his wife Martha (1927-1999) travelled to Prague in order to assess the situation there for Unitarians. The city’s large Unitarian congregation felt threatened by Hitler’s troops, as persecution under the Nazi rule was dramatically increasing. As their community continued to facilitate the flight of persecuted individuals, it became clear that sooner or later the Sharps themselves would become the next to be victimized, and they fled Czechoslovakia some months later.

Upon their return to the US, the Sharps joined an initiative to launch a rescue program in Europe, and one year later, in May 1940, as German troops occupied the north and west of France, the USC held its first meeting. It was constituted in Boston as an independent organization under Robert Dexter’s leadership.^23^ Its model paralled that of the Quakers’ American Friends Service Committee, but contrasted the Friends’ neutrality by openly supporting the Allied cause.^24^ For geostrategic reasons, the Sharps settled on Lisbon as the best location in Europe for the USC’s delegation to aid refugees in their transit to the Americas across the Pyrenees and Spain.

With the Armistice that led to the Vichy Regime, the French goverment multiplied the internment camps^25^ that took in thousands of refugees from different parts of Europe. These refugees ranged from Spanish Republicans and central-European Jews to Czech soldiers and international brigadists who had fought in the Spanish Civil War. The internment camps additionally hosted French Jews, most of whom were later deported to Nazi concentration camps. Living conditions in the camps declined as the war went on due to the lack of clothes, food, and medicine, in addition to human overcrowding and the poor physical state of the barracks. It was at this time that the USC’s general headquarters in Boston decided to prioritize direct humanitarian aid for the people in the camps, and this soon eclipsed their previous focus of helping singular fugitives flee to the Americas. This decision was informed by the legal obstacles and the high cost and risk involved in the individual rescues, among other reasons. It was assumed that the refugees’ situation in the camps could be improved by providing them with food, clothing, footwear, medicine, and sanitary materials. The USC became increasingly associated with direct medical aid, while the Quakers remained predominantly focused on the distribution of clothes and food to the refugees in internment camps. The USC even obtained special recognition by the Comité de Nîmes in 1941 and the ICRC in 1942 for its medical aid.^26^

As of June 1940, Marseille had become practically the last hope of those wanting to escape from Europe by sea. In this populous southern French port city and its surroundings, refugees from all over Europe crowded together, waiting weeks or even months for the indispensable visas that would allow them to embark for the Americas. It was in this context that the USC opened a second European relief office in Marseille, under the direction of the aforementioned Noel Field, a member of a Quaker family who became affiliated with the Communist Party after his experiences in the Spanish War.^27^ There, in collaboration with the Jewish Children’s Aid Society (*Oeuvre de Secours aux Enfants*), the USC set up a medico-social dispensary for refugees without resources, led by René Zimmer, an Alsacian refugee physician.^28^ The personnel of the dispensary was made up of health practitioners (physicians and nurses, but also pharmacists and dentists) as well as social workers. All were refugees and most were Jews. The Marseille dispensary, also known as the Clinique of Marseille, operated from July 1941 until 11 November 1942, when the Nazi army occupied all of France. Its services spanned surgery, otorhinolaryngology, dermatology, dentistry, orthopaedics, and pharmacy. It even had a laboratory, ultraviolet lamps, and a gymnasium. It carried out between 1,500 and 2,000 consultations every month, approximately one third of them pediatric.^29^ The clinic very effectively accomplished its mission to help refugees access medical attention and nursing care. It was especially helpful to restore its patients to good health, which was an indispensable requirement for obtaining a visa.^30^

After November 1942, the continuation of the Marseille dispensary on its previous terms was made unfeasible. Its activities could partly continue with the complicity of the Marseille city mayor, under the cover of a supposed municipal public health centre named the *Centre de dépistage et de prophilaxie*. The USC had to transfer its Marseille office to Geneva, where Noel Field and his wife Herta K. Field (née Vieser) carried on the task of coordinating the USC’s activities in France. They even organized support for the Resistance movements (the *Maquis*) on an individual and medical basis, arguing to the USC leaders that offering help to the Resistance fighters meant aiding the refugees.^31^ Some USC members entered France clandestinely to offer aid to the Resistance fighters, which meant that after the liberation they were able to immediately resume their pre-occupation activities, particularly those of the Marseille dispensary. They were able to recommence providing aid to all sorts of European refugees including Spanish Republicans, as well as providing medical attention to those recently freed from concentration camps who were suffering from malnutrition. The USC could now count on a splendid endowment, nearly half a million dollars between October 1944 and September 1945,^32^ from the US National War Fund, to implement its humanitarian programs in Europe.^33^

After the Normandy landings and the liberation of France in the summer of 1944, the JAFRC joined forces with the USC. Cannon accepted the honorary chairmanship of an appeal fund for the Boston chapter of the JAFRC at this time.^34^ The JAFRC agreed to hand over to the USC the task of channeling all the funds collected in the US to those most in need, not least because it was not licensed to distribute these private funds in Europe.^35^ The association between the USC and the JAFRC was decisive in setting up and further developing the Varsovie Hospital that was created at Toulouse following the failure of the so-called *Operación Reconquista de España* in October 1944. The *Operación* included an armed invasion of the Aran Valley at the Catalan Pyrenees with the aim of causing a popular uprising against Franco’s dictatorship, organized by the communist oriented *Unión Nacional Española* and carried out by exiled Spanish guerrilleros enlisted in the French Resistance.

Early in 1945, Cannon travelled with his wife to Mexico for three months. There lived Arturo Rosenblueth (1900-1970), his dearest former pupil, who had been his right hand man during his time at Harvard (1934-1944), as well as an assistant professor there. Rosenblueth extended an invitation to Cannon to join him on a visit to the *Sanatorio Barsky*.^36^ This clinic, named in honour of Cannon’s colleague, the former leader of the AMBASD, had been funded with donations from the US to look after Spanish Republicans. Cannon then wrote to Barsky in New York and Camille Soula (1888-1963), another physiologist colleague in Toulouse, to express his appreciation of their efforts to help the Spanish refugees relocated in Mexico. While in Mexico, Cannon may also have met the people who, alongside his Catalan pupils and colleagues in exile, made it possible for the Spanish edition of his book *The Wisdom of the Body* (1932) to be published in 1941.^37^

Cannon died in Boston in October 1945, six months after returning from his travels to Mexico. Because of his contribution to the organization of US humanitarian aid in the Spanish Civil War, the Varsovie Hospital added the designation of Walter B. Cannon Memorial to its name. This posthumous tribute made apparent the collaboration that had taken place between the USC and the JAFRC in this project, and was explained in the 1946 documentary *Spain in Exile* which devoted two minutes to the Varsovie Hospital. Both organizations were mentioned in the credits of the documentary, which showed in its sequences the dedication to the hospital written on a banner at the facade of the building.^38^ Yet, in February 1948 the USC withdrew its support to the hospital. Not only was the climate of political controversy unleashed by the campaign of the House Committee on Un-American Activities (HCUA) a factor, but also the USC had experienced a sustained decline in its funding from various sources (Unitarian congregations, the general public, and government funds) and was forced to cut the number of its programs in half.^39^ Moreover, the JAFRC had been charged, in the context of the McCarthyism offensive and according to the HCUA’s demands, with contempt of US Congress. In June 1947, all the members of its board, including the chairman Edward Barsky, were convicted, and after three years of appeals they would be sentenced to prison in 1950.^40^ In July 1948, the JAFRC inserted a statement protesting these convictions in the title-page of the first issue of the Varsovie Hospital journal to vindicate its relief work in favor of the Spanish refugees:

For many years we have been in charge of providing aid: medical aid, food and clothing to those Spanish Republicans who fought against Franco. We have established a hospital in the south of France, the Varsovie Hospital in Toulouse, and another in Mexico, the Barsky Sanatorium.

Thousands of men, women and children, who otherwise would have died, live today thanks to our work and our effort. Not one of the words uttered in the hundreds of pages of statements and accusations presented before the courts and before the “Anti-American” Committee come to change these facts. Not a single one of the accusations made against us, in the sense that we are not an aid agency for the aforementioned purposes, has been supported by facts.^41^

Thus, it is no wonder that despite the fact that the USC had been asking the JAFRC since early 1948 to remove the reference to Cannon Memorial from the name of the Varsovie Hospital, the JAFRC did not do it until about January 1950.^42^ At that time, the followers of most orthodox Stalinism condemning the independent policy of Yugoslavian Marshal Josip Tito imposed their will on the hospital leadership. Many Spanish communist militants in France, especially Catalans, were accused of being Titoists and bourgeois nationalists and thus purged or discharged from the party.^43^ A few months later, starting on 7 September 1950, the Varsovie Hospital became a victim of the *Opération Boléro-Paprika*. This was a police action directed and executed by the French Government, with the approval of General Franco, that aimed to curb the influence of communism by banning foreign communist parties and allied mass organizations in its dominions.^44^ The Varsovie Hospital was considered a bastion of the Cominform – the Information Bureau of the Communist and Workers’ Parties – in southern Europe so that it was stormed by the French police. Most of its doctors were arrested and expelled, via Corsica, to the Maghreb or to some Eastern European countries (Czechoslovakia and later Romania). Despite everything, the hospital managed to survive – even today it exists – mainly thanks to a group of French communist doctors led by Joseph Ducuing (1885-1963), professor of surgery and, for almost two decades, director of the *Centre Régional Anti-Cancéreux* (CRAC) in Toulouse.^45^

## The Religious Roots of Cannon’s Humanitarian Ethos: Science and Religious Dissent

In Anglo-American historiography there is an extensive tradition of studying the relations between science and religion, particularly with regard to scientific, medical, and technological innovations. Religious dissent in Great Britain and North America from the seventeenth to the twentieth century is included in these studies.^46^ This tradition can be traced back at least as far as Robert K. Merton’s 1936 doctoral dissertation. In it, he examined the relationship between Puritanism and the legitimation of science as a social activity in seventeenth-century England, a topic Charles Webster gave new life to forty years later in a brilliant monograph study.^47^

Within the Mertonian “happy marriage” of science and religious dissent, particular attention has been paid to the remarkable role played by two non-Puritan reformed churches whose religious concepts rely on parallel ideals. They are the Quakers and the Unitarians. The former took root in the late seventeenth century. The latter were *stricto sensu* from the late eighteenth century (1773 in London, 1776 in Edinburgh, and 1787 in Boston), though anchored in the Polish and Hungarian Socinianism of the second half of the sixteenth century.

Unitarians is a term originating in about 1570 that gradually supplanted previous designations for followers of the religious doctrine known as Unitarianism such as anti-Trinitarians, Arians, Racovians, and Socinians. Its sources of inspiration have not always been exclusively Christian. They may also have come from other religions such as Buddhism, Judaism, and Islam. To Unitarians, their individual conscience is the final authority for their own religious beliefs. They deny the doctrines of original sin and atonement, and in contrast, believe that all human beings have an innate worth and potential for holiness. They reject the orthodox doctrine of the Trinity and emphasize divine unity, the oneness of God. They believe Jesus to be entirely human rather than the son of God, and venerate him as a great soul and moral guide. They believe that the spiritual state of any person is dependent on that individual’s actions and personal responsibility. They affirm the essential unity of humankind and of creation. Among Unitarians’ most enduring values are a commitment to civil liberty and social justice, and an aspiration to overcome divisions and to unite all. Their openness to new ideas and their wish for interreligious dialogue has led Unitarians to champion the cause of universalism or “panreligionism” throughout the last two centuries, based on the assumption that all religions are different aspects of the same truth.^48^

Historians such as John Hedley Brooke and Geoffrey Cantor claim that dealing with the lives and experiences of individuals through biographical narratives is a more “appropriate genre for understanding the construction of science-religion relationships” than establishing correlations directly between scientific theories and the theological propositions of different belief systems.^49^

John Hedley Brooke has emphasized “respect for science and its promise of improvement” as, at least for a while, a central element of the Unitarian identity, something that might not be said of other factions of Christianity such as Anglicanism or Methodism.^50^ In his view, the “right to freedom of conscience on religious matters” – an essential feature of the Unitarian creed – has historically contributed to a comfortable relationship with scientific activity as a paradigm of free investigation. Moreover, Unitarians believe that “social progress is partly modelled on the scientific one” and that both scientific knowledge and social improvement flourish wherever there is a free exchange of ideas.^51^

## Cannon’s Religious Education

A biography of Cannon seems to be a favorable vehicle to decode the religious keys to his scientific and humanitarian ethos. A descendant of Irish immigrants from Ulster, he grew up in a strict Presbyterian household and was a member of a Congregational church. During his secondary studies at St. Paul High School (1888-1891), Cannon read Thomas H. Huxley’s (1825-1895) article in the *Nineteenth Century* journal (November 1885) where he claimed that, according to geological proofs, the Genesis account of the Creation was not true. Cannon then came across the polemics exchanged in the late 1880s to early 1890s between Huxley and the British politician William E. Gladstone (1809-1898) regarding Huxley's 1885 article. Cannon was deeply impressed by them and by the public controversies around them. This led him back to a previous controversy derived from exchanges between Huxley and the Anglican Bishop of Oxford, Samuel Wilberforce (1805-1873), some months after Darwin’s *On the Origin of Species* had been published in 1859.^52^ Cannon’s discovery of the implications of Darwinian thought for that great Victorian controversy led him to question his own “very strict Calvinistic ideas.”^53^

After openly confessing to abandoning his beliefs in the Congregational Church, despite the minister’s appeals to respect his own theological authority, Cannon considered himself entitled to an “independent judgement.”^54^ This religious crisis led him to new intellectual concerns and new interpersonal relationships. Particularly relevant was his friendship with the young Unitarian minister and preacher, Samuel McChord Crothers (1857-1927). Crothers had just arrived at St Paul High School after training at Harvard and going through a similar religious experience. Crothers’s sermons in favor of the “freedom of the human soul to think for itself so long as such action did not interfere with the freedom of others” helped Cannon to stand firm in his principles and resist outside pressures.^55^ In 1893, one year after Cannon had entered Harvard College, Samuel Crothers moved to Harvard with his wife to be a minister of the Cambridge Unitarian Church. Their family house would become a refuge for Cannon, and his friendship with the couple would last a long time.^56^

Cannon's religious crisis, as well as his stimulation by reading works by authors such as the Catholic bishop of Chicago and advocate of higher education John L. Spalding (1840-1916), the influential British Unitarian religious philosopher James Martineau (1842-1910), and the US Unitarian theologian James Freeman Clarke (1810-1888), led Cannon to be concerned with the foundations of Christian doctrine. Huxley’s polemical writings had piqued Cannon’s interest in the works of other authors who were to be particularly influential in his decision to pursue a university education. These included theorists and popularizers of evolutionary thought such as Herbert Spencer (1820-1903), John Tyndall (1820-1893), George H. Lewes (1817-1878), John Fiske (1842-1901), and Darwin himself.^57^

## Harvard Unitarianism: The “Religion of the Future” of Charles W. Eliot

Cannon’s decision to enter Harvard was encouraged by May J. Newson, his English teacher at St Paul High School, who had studied there. Cannon completed his Bachelor of Arts studies at Harvard College (1892-1896) before studying Medicine at the Harvard Medical School (1896-1900). Founded in 1636, Harvard College was initially aimed primarily at training Congregationist and Unitarian clergy. Influenced by the Enlightenment, the College underwent a change in the early nineteenth century. As the Harvard senior professorship and presidency were captured by liberal Unitarians around 1804-1806, the College’s ideologically traditional orientation pivoted to and was increasingly dominated by liberal religious ideas.^58^

The educational principle of intellectual freedom was finally established at Harvard thanks to the curricular reform promoted by Charles W. Eliot (1834-1926). Eliot was a chemistry professor who held Harvard’s presidency for forty years, from 1869 to 1909. As a result of his presidency, compulsory teaching of religion and the pre-eminence of Christian doctrine within the elective theology lectures were suppressed.^59^

Rather paradoxically, the educational reform which fueled the secularization of Harvard’s higher education had theological roots. Eliot’s views were inspired by the liberal Unitarian beliefs of the preacher William Ellery Channing (1780-1842) and those of the transcendentalist essayist Ralph Waldo Emerson (1803-1882). Transcendentalism, the dominant religious movement in early nineteenth-century Boston, developed as a parallel movement to the ideas introduced by the Unitarians. This movement emphasized freedom of conscience and the value of intellectual reason and was closely linked to the Romantics, though without opposing the empiricism of science. Transcendentalists were strong believers in personal freedom and the power of the individual, and they longed for a more intense spiritual experience that would extend beyond traditional Unitarians’ sobriety, mildness, and calm rationalism. Thanks to the Transcendental Club in Cambridge, Massachusetts, founded in 1836 by Emerson among others, the movement became more coherent and gave rise to Transcendentalism as it is known today. There were three essential principles on which Eliot built his educational reform: the “natural dignity and worth of man” (shared by both of his inspirers, Channing and Emerson), the “right of private judgement unrestrained by authority and unfettered by creeds” (derived from Emerson), and the “original, unbroken, and universal community of nature, man, and God” (taken from Channing). Though he was not theologically innovative, Eliot became a radical innovator of higher education by applying these theological principles to its reform.^60^

According to Eliot’s pragmatic religious ideal, “true Christianity” was not “a body of doctrines, nor an official organization to direct and control men’s minds and wills,” but “a way of life.” Eliot’s “this-world focus within religion” had “the whole world for the field of the loving labors of its disciples,” where “its fundamental principle of serviceableness” admitted “an infinite variety and range in time and space.” This meant that to him religion overstepped “the realm of mere individual morality” and went into the “arena of social responsibility,” as its purpose – coincidentally aligned with the purpose he assigned to higher education – was to “produce people who possess the type of character that compels them to social service.”^61^ To Eliot, this “twentieth-century religion” called to become “the religion of the future” was

… not only to be in harmony with the great secular movements of modern society – democracy, individualism, social idealism, the zeal for education, the spirit of research, the modern tendency to welcome the new, the fresh powers of preventive medicine, and the recent advances in business and industrial ethics – but also in essential agreement with the direct personal teachings of Jesus as they are reported in the Gospels.^62^

Eliot’s views of the “religion of the future” may well have influenced Cannon’s humanitarian ethos, and may be crucial to understanding it.^63^

## Cannon’s Most Influential Masters at Harvard

Among his influences at Harvard College, Cannon highlighted three of his lecturers as having altered the course of his life. The first of these was the eugenicist and biologist Charles B. Davenport (1866-1944), with whom he made his first biological investigations. The second was the zoologist George H. Parker (1864-1955), who was interested in the anatomy and physiology of the sensory organs and animal reactions, and to whom he was student assistant for two years. The third was the philosopher, psychologist, and physician William James (1842-1910). The “freshness and constant unexpectedness of [James’s] ideas and … phrasing of them” fascinated Cannon, and James allegedly pragmatically dissuaded Cannon from devoting himself to philosophy.^64^ The way to James may have been paved by Cannon’s previous reading at St Paul High School of the essay “The Ethics of Belief” (1875) by the mathematician and philosopher William K. Clifford (1845-1879). In his lecture “The Will to Believe” (1896), James responded to Clifford’s views on evidentialism, faith, and overbelief.^65^

In the fall of 1896, Cannon entered Harvard Medical School, receiving his medical degree there in 1900. Despite his early involvement in experimental physiology, it appeared he remained for a long time determined to become a medical practitioner, emphasizing the relevance of applying physiological research to solving clinical problems.^66^ Yet, at the end of his medical studies, Cannon chose to devote himself entirely to physiology. His professional career in this field developed at Harvard Medical School. There, he was first instructor (1900-1902), then assistant professor (1902-1906). Finally after his predecessor and master, Henry P. Bowditch (1840-1911), retired he became the George Higginson Professor of Physiology (1906-1942). At the end of his long and fruitful career, Cannon summarized his research concerns as having passed from an initial attention to “digestive functions, through studies of emotional effects on bodily processes, to regulation of steady states in the body and the chemical mediation of nerve impulses.”^67^

There were two framed prints hanging on the wall of Cannon’s office at Harvard Medical School. One was an image of Claude Bernard instructing a group of students, and the other, a portrait of Charles Darwin as an elderly man. The noteworthy presence of these prints strongly suggests that the two scientists were the major models for Cannon’s research career.^68^ He recognized Claude Bernard (1813-1878) as the main creator of the experimental physiological method and the formulator of the concept of *milieu intérieur*, on which Cannon built the concept of homeostasis.^69^ He felt quite attached to Bernard, as they shared a determination to use vivisection as their method of experimental laboratory research, and shared the belief that it was the quintessential method for such purposes. Cannon’s convictions in this respect led him to preside for seventeen years (1908-1925) over the physiology and pathology section of the American Medical Association (AMA) during the most crucial period of antivivisectionist agitation in North America. At that time, Cannon confronted antivivisectionists by arguing that their aims, “misconceptions,” and methods were a threat to “freedom of medical investigation” as well as a danger for the population as a whole.^70^ In the early autumn of 1905, he gave an address on the ideals of science to the national Conference of Unitarian and other Christian Churches, with the dual purpose of popularizing science, and persuading his audience to support medical scientists in the face of antivivisectionists’ hard attacks.^71^

Reference has already been made to the crucial impact that the great Victorian controversy around the theory of biological evolution had on Cannon’s teenage religious crisis. Darwin’s influence was clearly present in many aspects of his work, for instance, in the “emergence theory of emotions” that Cannon developed alongside his pupil Philip Bard (1898-1977). Darwin’s commitment to monogenism, a theory of human origins holding a common descent for all human races, and his anti-slavery convictions, Darwin’s “sacred cause,” were apparently related to his family connections with Unitarianism (from his mother and Wedgwood cousins, at least).^72^ Darwin’s ethos on this matter seems analogous to the one underlying Cannon’s internationalist commitment to freedom and democracy in times of totalitarianism and worldwide war.

## Links of Cannon’s Relatives to Unitarianism

There are clear indications of the relevance of Unitarianism within the family life of the Cannons. Walter's wife Cornelia James Cannon (1876-1969) came from a family with Unitarian tendencies, which included an admiration for New England transcendentalism, evident in their careful reading of Ralph Waldo Emerson and Henry David Thoreau. At Harvard, Cornelia, like Walter, took refuge in the safe haven provided to all outsiders by the Unitarian minister Crothers and his wife.^73^ Having been, like her sisters, involved early on in the Women’s Christian Temperance Union, she was a reformist, feminist, and eugenic writer. She was socially active in the fights for women’s suffrage, birth control, and immigration policies, all causes esteemed by the Unitarians. The public image of the Cannons was by no means alien to the strong personality and the social and political activism commitments of Cornelia. Actually, her influence may be guessed in Walter’s public inroads, albeit always from an academic biomedical perspective, in areas such as sex and reproduction policies (from 1918 for the Committee for Research in problems of Sex), and alcoholism (in 1939 for the Research Council on Problems of Alcohol).^74^

His sister Ida Maud Cannon (1877-1960) trained and worked as a visiting nurse before enrolling at the new Boston School for Social Workers, as encouraged by Cornelia. Ida and her sister Bernice lived in their brother’s household in Cambridge for 27 years. Ida pioneered the medical social work movement, and she greatly contributed to the development of its theory and practice from the Massachusetts General Hospital where she worked for nearly forty years after being recruited by Richard C. Cabot (1868-1939). A remarkable Unitarian, physician, philosopher, educator, and social work pioneer, Cabot was one of the medical leaders of the Emmanuel Movement, a psychologically-based approach to religious healing spread in 1906 from the Emmanuel Episcopal Church in Boston and tending to play down the relevance of the religious element,^75^ in which Ida Cannon may also have been involved in her early years at the hospital. Cabot was also the source of inspiration for the idea of hospital and dispensary social work that she applied to her work at the hospital. Ida’s identification with “progressive movements for public health and social welfare” – she wrote *Social Work in Hospitals: A Contribution to Progressive* *Medicine* – did not prevent her from being a faithful Unitarian who understood religion to be a way of living in the image of Christ. Quite the opposite – she applied her faith to her daily work, claiming that every person’s duty was “to find their own vocation and make a unique contribution to the world.” Allegedly, Ida had embraced Unitarianism by influence of Walter, and believed, like her older brother, that “spiritual exploration and scientific research could not yield meaningful knowledge if pursued independent of one another.”^76^

Finally, Marian Cannon Schlesinger (1912-2017), one of the four daughters of Walter and Cornelia Cannon, referred in her memoir to several episodes of their family life in Boston that show the density of its Unitarian atmosphere. For example:

It was a jolly mix [of kids], but seemed not to have affected the general tone of our high school life. We were all outsiders thrown together higgledy-piggledy and the rich mélange of personality and cultures was an education in itself. We did not think much in ethnic terms in those days. Differences in background and even in religion were remarked upon but taken for granted, by them and by us. I can, however, remember us as grammar school children being little Unitarian skeptics, once baiting our Catholic playmates with questions as to the validity of the Virgin Birth, referring to what we thought were appropriate passages in the Bible to prove our point. There must have been talk around the house on the subject as the result of the Scopes Trial in Tennessee [or the Scopes Monkey Trial, the most well-known legal case in the history of the controversies between Creationism and Evolutionism apropos of Darwin’s *On the Origin of Species*], for my mother wrote, "The children actively discuss the Virgin Birth, thanks to the Fundamentalist’s gift of the subject to polite conversation. They thought it very illogical that God should be both God and Christ." But it was all rather like the blind leading the blind. I doubt that any of us knew what "Virgin Birth" means, and we won the argument not through superior theological authority or mastery of esoteric doctrine but because they were playing in *our* back yard and there were more of us!^77^

Thus, it is no surprise that Cannon’s widow willingly accepted the USC’s initiative of adding the surname Walter B. Cannon Memorial to the name of the Varsovie Hospital in Toulouse in 1946. It also makes sense that she requested that Cannon’s name be removed from it once the USC had withdrawn its support to the hospital in February 1948.

## Science, Democracy and Liberty: Cannon’s Major Political Causes and Ideology

Given the views of his greatest influences, it is no wonder that Cannon so strongly stressed throughout his work that the connections “between democracy and science” were as obvious as those “between liberty and democracy.” On one hand, social evolutionary views led him to argue that “modern democracy” evolved alongside industrialization and urbanization. The multitude of devices that could now be produced industrially thanks to “scientific research and experimenting and inventing” required the concentration of “people together in populous cities and required varieties of skill and intelligence in work on technical procedures.” Increasing population density meant that “points of contact between individuals and groups multiplied and opportunities for concerted thinking and action for common interests greatly increased” so that “free popular education, public hospitals, [and] protection of community health” were among the “natural results” of the “democratic process.”^78^

On the other hand, Cannon affirmed the “liberty of the individual” but he recognized the “essential organic relations of the individual to the common body politic” which led him to claim that what an individual “does for public welfare has values for all the members of the social order, himself included.”^79^ Certainly, he had already referred to the parallels between biological and social regulation mechanisms in the epilogue “Relations Between Biological and Social Homeostasis” that he had added to *The Wisdom of the Body* (1932) at the suggestion of his wife Cornelia.^80^ In December 1940, Cannon went even further, claiming that the body politic of human societies was ideally modeled on the organization of the human body. He introduced this idea in his speech “The Body Physiologic and the Body Politic,” which he gave at the end of his year as president of the prestigious American Association for the Advancement of Science (AAAS).^81^

Moreover, in explicit agreement with the philosopher Ralph Barton Perry (1876-1957), Cannon considered that democracy was a fragile and perpetually perfectible good. A pupil of William James, Perry was a member of Cannon’s circle of friends at Harvard – most of whom were not linked to medical research – who had founded the Wicht Club. This was an organization where young scientists and philosophers, united by a common interest in scholarly inquiry and scientific ideas, met informally on a monthly basis. Following Perry, who he considered to be one of his “two closest and dearest friends,” Cannon denied claims that “the democratic ideal and its attendant freedom have been nearly achieved.” To him, no democratic achievement could be taken as “fixed and secure” because of the continued existence “of unwarrantably privileged classes, of hopeless economic groups, and of powerful selfish interests which press heavily for especially favorable legislation.”^82^ Perry’s views might have influenced Cannon’s social organicism as his thoughts on this matter were far from the concept’s classical nineteenth-century roots. Indeed Cannon thought that the “body politic” manifested itself “unable to equal the perfect regulation of the body biologic.”^83^

The relations between science and democracy were at the heart of the scientific community in the late 1930s, when a large number of scientific academics gravitated towards liberal and leftist activism. These scientists were concerned about the lasting political and social effects of the Depression and the advance of fascism, for which the Spanish Civil War was the focal point. In the US, these scientists pushed the policies of the AAAS towards a new social vision. This was done by tacking, in a series of “Science and Society” symposia, not only various aspects of the social impact of science, but also the issue of the social responsibilities of scientists. These symposia were held on the occasion of the AAAS’s semiannual meetings in December 1937. In 1938, organizations arose such as the Lincoln’s Birthday Committee for Democracy and Intellectual Freedom (LBCDIF), as well as the more radical American Association of Scientific Workers (AASW). The LBCDIF was an antifascist organization of scientists founded by the anthropologist Franz Boas (1858-1942). It became better known under the name of the American Committee for Democracy and Intellectual Freedom (ACDIF). The ACDIF and the AASW later joined forces to further the social and political goals of liberal and left-wing scientists. They mostly worked towards a better organization and application of science for the benefit of the common good. They also promoted the notion that the popularization of science would act as a rational antidote to fascism and to Nazi racial theories.^84^

To Cannon, an interest in the relations among science, democracy, and liberty proved indispensable to his role as scientific investigator. As his professional pursuit depended “immediately on the liberty which a democratic government most reliably provides,” it comes as no surprise that he was deeply involved in the science and society movement. As the influential AAAS’s 1939 president, he was one of four distinguished academics who broadcast a radio warning to the public under the sponsorship of the LBCDIF. They urged the public to safeguard democracy and condemn totalitarian governments (mainly those of Germany and Italy) for stifling science. In the context of the Nazi-Soviet pact of August 1939, Cannon was one of more than 500 signatories of a national Peace Resolution promoted by the AASW. This resolution aimed to dissuade President Roosevelt from involving the US in the coming conflict. It claimed that the “continuance of progress now largely depends upon the scientists of neutral nations,” and that “American scientists can best fulfill their share of this responsibility if the United States remains at peace.” Yet, after the Nazi invasion of Belgium and France in May 1940, Cannon publicly withdrew his signature from that manifesto, and in early June he renounced his sponsorship of the Boston chapter of the AASW.^85^ On 7 June, the day after his resignation, Cannon wrote in a private letter to his colleague Anton Julius Carlson (1875-1956), then the president of the AASW,

There is evidence of a group here in Boston which has been aggressively arranging for dominance of the [A.A.S.W.] chapter and which is known to be of the extreme left persuasion. Ructions have resulted from the action of this group and members of the Association have resigned in consequence of these high-handed practices.^86^

This did not prevent Cannon from acting in medical solidarity with the Soviet Union from 1943 onwards, after its invasion by German troops. He gave his support through the American-Soviet Medical Society, which had been founded under the auspices of the prominent professor of medical history at the Johns Hopkins University Medical School, Henry E. Sigerist (1891-1957). Because he was the only American medical member of the Academy of Sciences of the USSR, and because of his genuine concern for Soviet science and medicine, Cannon became the first president of the American-Soviet Medical Society.^87^ As Sigerist would stress on the occasion of the memorial held for him at the New York Academy of Medicine in October 1945, Cannon “had the courage, vision, and the imagination to be and to call himself openly an anti-fascist at a time when it was not popular at all and when it was courageous for a man to oppose Mussolini and Hitler.”^88^ Yet Cannon’s solidarity involvement was motivated, on the one hand, by his admiration towards the social status Soviet scientists had been granted, which contrasted the lack of public support for US universities during the Great Depression. On the other hand, Cannon was curious as an “experimenter” about the humanist “experiment” being performed in the Soviet Union, despite his increasing concern from 1935 onwards about the authoritarian tendency of its government.^89^

As Cannon’s contradictory movements regarding the AASW’s 1940 Peace Resolution plainly reveal, he began to keep his distance from the Soviet government as it drifted towards authoritarianism. At that historic crossroads, Cannon appeared to have chosen “democratic socialism” like many of the American academic scientists of his generation. In 1936, his predecessor as AAAS president, Edwin G. Conklin (1863-1952), claimed that this ideology “best preserve[d] the balance between individual freedom and social regimentation and represent[ed] the ‘scientific’ solution to the problem” by spreading the “spirit of science and the method of science…to society and government.”^90^

Examinations of Cannon’s political ideology, and of whether or not it could be considered left-wing, would be incomplete without dealing with some authors’ interpretations of it. On one hand, his son-in-law Arthur Meier Schlesinger (1917-2007) strikingly portrayed him as a man “rather conservative, mistrustful of the too-smooth FDR [Franklin Delano Roosevelt] and his too-certain New Dealers” to the point of having “voted for Alf Landon,” the Republican Party’s nominee in the 1936 presidential election. Schlesinger pointed out that Cannon’s enthusiastic partisanship of the Spanish Republic had “gained him an underserved reputation as a radical, if not a red, which he endured stoically.” He claimed that it derived from his visit to Spain and his friendship with the Spanish physiologist Juan Negrín, who was one of the leaders of the Spanish Socialist Party (PSOE).^91^ While Schlesinger’s comment provides clear evidence against Cannon’s contemporaries who denounced him as a communist, Cannon’s republican instinct requires some explanation. It might be related to the fact that before the 1932 presidential election of Roosevelt, the Republicans – and not the Democrats – were the progressive party. It may even be more specifically related to Cannon’s devotion to the Republican Lincoln and his major role in the abolition of slavery in the US. Moreover, the fact that New Deal policies did not mitigate the serious impact of the Great Depression on scientific research and employment reinforced “the nascent doubts of many scientists about fundamental aspects of the American political and economic system.”^92^

On the other hand, according to Anne-Emanuelle Birn and Theodore Brown, the AMBASD and the NACASD’s mobilization in solidarity with the Spanish Republic must be framed in the context of a “health internationalism” that was based on social medicine as much as on proletarian internationalism. These movements were both part of the fight for equality for all human beings and for the emancipation of all oppressed people. Moreover, they argue that the fact that Cannon’s activism during the Spanish Civil War might be considered counter-hegemonic does not imply that his political convictions were, in principle, left-wing ones in the context of the US in the 1930s.^93^ However, in the face of the relentless advance of fascism in Europe during the 1930s and early 1940s and the tragedy of the Spanish Civil War, many contemporary American and British scientists’ political ideologies pivoted toward leftism. Many of them did in fact become communists, while no few others turned towards democratic socialism, as Cannon did.^94^

## Concluding Remarks

Almost thirty years ago, the historian Saul Benison (1920-2006) wondered about the reasons behind Cannon’s political beliefs and concerns in the troubled times he lived in.^95^ Benison discarded views considering him “a socialist” (Peter Kuznick) or a “social theorist” (Stephen J. Cross and William R. Albury).^96^ He concluded that “Cannon was first and above all a physiologist,” and that his politics were defined by “his concern for protecting the freedom necessary for the development of science.” He claimed that Cannon’s politics were “not bound by political ideology or geography but by a belief in the enrichment of the human spirit through science, a conviction that made his politics that of a citizen of the world.” More recently, Mathieu Arminjon, who has sharply analyzed his “biocracy” theory, has claimed that Cannon appears to have “embraced a general humanism, rather than defending a particular political model.”^97^ These understandings of Cannon’s deep, lifelong political commitment to various humanitarian causes seem incomplete because they fail to take into account the religious roots of his humanitarian and scientific convictions. In other words, it might be the case that if historians knew more about Cannon’s Unitarian ethos, they would not have given misrepresented his readings.^98^

Particularly striking is Benison’s complete omission of Cannon’s Unitarian beliefs, which are entirely absent from the pages of the extensive two-volume biography he wrote jointly with A. Clifford Barger and Elin L. Wolfe.^99^ Their broad and expansive indexes fail to make even a single reference to Unitarianism or the Unitarian Church, yet often mention Cannon’s concern for Spanish refugees in France with numerous references to it from the very moment of the *Retirada* and the great exodus across the Catalan border in the winter of 1939.^100^ Nor do they mention the Varsovie Hospital in Toulouse, or the honorific addition of Walter B. Cannon Memorial to its name in 1946, shortly after Cannon’s death, as a tribute to him and the work he did there. One reference is made to the Unitarian Service Committee (USC) in Benison, Barger, and Wolfe’s long study, but this reference is stripped of any mention of the direct link between Cannon and this humanitarian organization. The USC is reduced to merely being an association through which the JAFRC channeled its aid to the Spanish Republican refugees in France.^101^

Cannon does appear to have avoided any reference to the Unitarian Church or any organization of its kind, including the USC, in his publications. In the case of his autobiography, Cannon’s omission could be excused by his alleged purpose of dealing only with one of his many “social selves,” that of a “scientific worker.”^102^ Yet his absolute silence in this respect may rather be ascribed to deliberate discretion, in keeping with his presumed sense of privacy and abhorrence of any religious proselytizing, one of the principles of Unitarianism. Cannon’s biographers’ avoidance of the religious and most openly political dimensions of his self indicate a decision to confine themselves to the self-imposed limits of his autobiography.^103^

The most conclusive evidence that Cannon was a long-serving, faithful member of the Unitarian church lie in the last words of a plain editorial note published fifteen months after his death. This note, published in the *Newsletter* of the “First Parish in Cambridge,” read, “Dr. Cannon was a member of our congregation and a pew holder for many years.”^104^ The emblematic “First Parish,” the earliest Unitarian church in the Massachusetts town, had been closely involved in the foundation and development of Harvard University as well as in the shaping of remarkably liberal religious thought in the Cambridge Congregation within the American Unitarian Association. This Unitarian parish could not turn a blind eye to the tragedy of the Spanish Civil War and the increasingly worrying situation in Europe during the late 1930s. Its Social Service Committee of twenty years was replaced, after its disbanding in 1936, by a new Social Relations Committee, which Ida Cannon helped found, representing “a shift in the priorities of the congregation away from social service *per se* and toward promoting world peace and protecting civil liberties.”^105^

This evidence of Cannon's Unitarian faith reveals not only the extent to which his scientific, political, and religious ideals were in conjunction, but also divulges the inspiring role that Cannon's memory played in New England during the early Cold War years regarding the fate of the Spanish Republican refugees in France. In mid January 1947 the First Parish’s *Newsletter* echoed an urgent appeal just launched by the Walter B. Cannon Memorial Hospital Fund for Spanish Republicans in Southern France in Boston to raise funds for sustaining the Varsovie Hospital in Toulouse under the headlines, “They fought 2 wars for you, now ask you for life.” Its contents particularly focused on Cannon, paying him tribute as a “great scientist and humanitarian,” and “active friend of the Spanish Republicans from the very beginning.”^106^ Fully reproduced in the *Newsletter*, the campaign was co-chaired by Frederick May Eliot as president of the AUA,^107^ the Harvard medical professor and Nobel Prize laureate in medicine George R. Minot, the Harvard professor of philosophy and member of Cannon’s circle of Harvard friends Ralph B. Perry, and an attorney named Paul T. Smith.

In sum, the present study has shown how important is to branch out in our studies of medical and scientific practice to include practitioners’ broader social and religious communities in order to understand their motivations, achievements, and behavior.

## Funding

This contribution is the result of ongoing research within the framework of the projects funded by the Spanish State Research Agency (AEI): “Relief action and medical technologies in humanitarian emergencies, 1850-1950: agencies, agendas, spaces, and representations” (HAR2015-67723-P), and “Transnational humanitarian medicine and technological innovation in spaces of confinement 1870-1950” (PID2019-104581GB-I00). We are indebted to Anne-Emanuelle Birn, Andrew Cunningham, Javier Fernández Galeano, Michael R. McVaugh, Maica Pérez-Aguado, Emma Sallent Del Colombo, Jaume Sastre and the three anonymous referees for their comments and criticisms, and to Espie Krementsova (Canada) for her valuable revision of the English style.

## Footnotes

1 Allan M. Brandt and Martha Gardner, “The Golden Age of Medicine?” in *Companion Encyclopedia of Medicine in the Twentieth Century*, ed. Roger Cooter and John Pickstone (London: Routledge, 2003), 21-37.

2 Elin L. Wolfe, A. Clifford Barger, and Saul Benison, *Walter B. Cannon: Science and Society* (Cambridge, MA: Harvard University Press, 2000), 354-375.

3 On the USC’s activities in Europe during the Second World War, see Susan Elisabeth Subak, *Rescue and Flight: American Relief Workers Who Defied the Nazis* (Lincoln: University of Nebraska Press, 2010). On the JAFRC, see Sebastiaan Faber, "Image Politics: U.S. Aid to the Spanish Republic and its Refugees," *Revista Forma* 14 (2016): 21-34.

4 Àlvar Martínez Vidal and Alfons Zarzoso Orellana, “Un hôpital moderne dans le sud de la France pour les réfugiés espagnols: l’Hospital Varsovia/Walter B. Cannon Memorial (1944-1950),” in *L’Hôpital Varsovie. Exil, médecine et résistance (1944-1950)*, ed. Àlvar Martínez-Vidal (Portet-sur-Garonne: Loubatières, 2011), 9-38, at 14-15; Rosa Toran and Àlvar Martínez-Vidal, *El metge Josep Torrubia Zea. Lliurepensador, maçó i socialista* (Catarroja: Editorial Afers, 2021), 183-210.

5 Linda Palfreeman, *Spain Bleeds: The Development of Battlefield Blood Transfusion during the Civil War* (Eastbourne: Sussex Academic Press, 2015); Sebastian Browne, “Blood Propaganda,” in *Medicine and Conflict: The Spanish Civil War and its Traumatic Legacy* (London: Routledge, 2019), 112-158.

6 Linda Palfreeman and Peter Pinkerton, “Transfusion in the Spanish Civil War: Supply and Demand, the Role of the ‘Blood Transfusion Officer’ and British Planning for the Outbreak of the Second World War,” *Transfusion and Apheresis Science* 58 (2019): 102671.

7 The *Junta de Defensa Passiva de Catalunya* built more than one thousand shelters against bombing and chemical attacks. See Fátima Martínez Lopezosa, *La defensa passiva a Barcelona durant la Guerra Civil: Els refugis antiaeris*, Universitat de Barcelona, Bachelor Degree Dissertation supervised by Joan Villarroya Font, 2013-2014. Further, on Barcelona air-raid shelters and their influence on WWII in England, see Jaume Valentines-Álvarez, Guillermo Lusa, and Antoni Roca-Rosell, ed., *El Fons “Ramon Perera”: Imatges de la defensa passiva a Catalunya, 1938-1939* (Barcelona: Càtedra UNESCO de Tècnica i Cultura, UPC, 2008); Jaume Valentines-Álvarez, “Tecnologías para sobrevivir la violencia total, 1936-45. Tragedia de refugios antiaéreos en tres actos, prólogo y cuadro final,” in *Violência política no século XX. Um balanço*, ed. Ana Sofia Ferreira, João Madeira, and Pau Casanellas (Lisboa: Instituto de História Contemporânea, 2017), 497-507.

8 Gabriel Pretus, *Humanitarian Relief in the Spanish Civil War (1936-1939): The Independent and Non-Partisan Agencies*, Royal Holloway University of London, Department of History, Doctoral Dissertation, 2011, 104-126. There is an English edition (Lewiston, NY: Edwin Mellen Press, 2013) and a Spanish one (Albolote, Granada: Comares, 2015) of this PhD thesis.

9 On Cannon’s relationships with Spanish physiologists, and his support of the Spanish Republic during the Civil War and its aftermath, see Miguel Marco, “Spanish Influence in the Life and Work of Walter B. Cannon (1871-1945),” *Neurosciences and History* 2 (2014): 132-139; Carmen Pérez-Aguado, Alberto de Leiva-Hidalgo, Alejandra de Leiva-Pérez, Eulalia Brugués, “Walter B. Cannon: A History of Commitment,” *Vesalius* 26 (2020): 218-231.

10 Walter B. Cannon, *The Way of an Investigator: A Scientist’s Experiences in Medical Research* (New York: Norton, 1945), 161; Wolfe, Barger, and Benison, *Cannon: Science and Society*, 354-375.

11 Cannon, *Way of an Investigator*, 161-162.

12 Ibid.

13 Phillip Deery, “The Doctor: Edward Barsky,” in *The Red Apple: Communism and McCarthyism in Cold War New York* (New York: Fordham University Press, 2014), 11-38.

14 Walter J. Lear, “American Medical Support for Spanish Democracy, 1936-1938,” in *Comrades in Health: US Health Internationalists, Abroad and at Home*, ed. Anne-Emanuelle Birn and Theodore M. Brown (New Brunswick, NJ: Rutgers University Press, 2013), 65-81; Jane Pacht Brickman, “Medical McCarthyism and the Punishment of Internationalist Physicians in the United States,” in *Comrades in Health*, 82-100. On Fredericka Martin, see Barbara Martin, “My Cousin Fredericka Imogene Martin,” *The Volunteer* 35 (2018), https://albavolunteer.org/2018/11/my-cousin-fredericka-imogene-martin-by-barbara-martin/.

15 Eric R. Smith, *American Relief Aid and the Spanish Civil War* (Columbia, MO: University of Missouri Press, 2013), 90-93, 104, 108.

16 On Negrín, see Enrique Moradiellos, *Negrín. Una biografía de la figura más difamada de la España del siglo XX* (Barcelona: Península, 2006); Gabriel Jackson, *Juan Negrín: Physiologist, Socialist and Spanish Republican War Leader* (Brighton: Sussex Academic Press, 2010).

17 A fervent Catholic, but loyal to the Republic, Jesús M. Bellido was a chairman of Pharmacology at the University of Barcelona and a representative of the so-called “Catalan biological school” whose members mostly went into exile after the Spanish Civil War. On 20 January 1939, just six days before the city was seized by Franco’s troops, he and other scholars promoted the designation of Cannon as *honoris causa* doctor of the University of Barcelona. This connection led Cannon to be most worried about Bellido’s fate after the Nazi invasion of France in May-June 1940. Cannon was particularly concerned about rescuing him since Republican leaders exiled in France – such as Lluís Companys, Joan Peiró, and Julián Zugazagoitia – were being arrested, deported to Francoist Spain, and executed. He was alarmed about not receiving any response to his letters to Bellido and the letters not being returned to him, either. Despite their mutual esteem, they appear to have never talked each other about their religious convictions. Nor has any allusion to religious elements in the correspondence between Cannon and his pupil Rossend Carrasco i Formiguera (1892-1990) been detected, even though Carrasco was the brother of a liberal Catholic, founder of the *Unió Democràtica de Catalunya*, who was executed by Franco in April 1938. See Harvard Medical School, Countway Library of Medicine (CLM), Boston, MA, Cannon’s Papers. Correspondence between Walter B. Cannon and Jesús M. Bellido; Correspondence between Walter B. Cannon and Rossend Carrasco (CLM. Cannon’s Papers. H MS, c 40, Box 46, Folder 598); Correspondence between Walter B. Cannon and Jaume Pi-Sunyer (CLM. Cannon’s Papers. H MS, c 40, Box 46, Folder 603); Thomas S. Glick, “Walter B. Cannon i la ciència catalana d’entreguerres,” *Butlletí de la Societat Catalana de Ciències Físiques, Químiques i Matemàtiques* 4 (1985): 139-157; Wolfe, Barger, and Benison, *Cannon: Science and Society*, 604 and n. 23; Àlvar Martínez Vidal and Emma Sallent Del Colombo, “‘Making Science Understandable’. Divulgació científica i mediació editorial: el cas de *The wisdom of the body* de Walter B. Cannon (1871-1945),” *Actes d’Història de la Ciència i de la Tècnica* 2 (2009): 45-59; Àlvar Martínez Vidal and Emma Sallent Del Colombo, “Ciencia en el exilio, una forma de resistencia. La traducción castellana de *The Wisdom of the Body* de Walter B. Cannon (México, 1941),” *Cultura Escrita & Sociedad* 10 (2010): 149-175.

18 Cannon, *Way of an Investigator*, 162.

19 Faber, “Image Politics.”

20 Haim Genizi, “Christian Charity: The Unitarian Service Committee’s Relief Activities on Behalf of Refugees from Nazism, 1940-5,” *Holocaust and Genocide Studies* 2 (1987): 261-276.

21 On Robert Dexter’s activities, see Subak, *Rescue and Flight*, xvii, *passim*.

22 On the Unitarian Service Committee, see ibid., 26-28, *passim*. On the activities of the USC’s Marseille office, see Aurelio Velázquez-Hernández, “The Unitarian’s Service Committee Marseille Office and the American Networks to Aid Spanish Refugees (1940-1943),” *Culture and History Digital Journal* 8 (2019), https://doi.org/10.3989/chdj.2019.021; Jon Arrizabalaga, “La asistencia médica a los refugiados en Francia durante la Segunda Guerra Mundial: la Clínica de Marsella (1941-1945),” *Dynamis* 40 (2020): 67-91.

23 Subak, *Rescue and Flight*, 25-26.

24 Genizi, “Christian Charity,” 265.

25 Although the usual denomination at the time was that of “concentration camps,” French historiography distinguishes between “internment camps” that were devoted to lodging refugees in better or worse conditions, and the “concentration camps” themselves that were rather intended for forced labor and annihilation. See Denis Pechansky, *La France des camps. L’internement, 1938-1946* (Paris: Gallimard, 2002).

26 American Unitarian Association, S*aving the Future in Europe: The First-Hand Story of the Unitarian Service Committee’s Medical Work in Unoccupied France*. Boston, 1942, folder bMS 16035/1 (4), [4], Unitarian Service Committee Archives [USCA], Andover-Harvard Theological Library, Cambridge, MA; Genizi, “Christian Charity,” 263-64; Pechansky, *La France des camps*, 249, 254. The Committee of Nîmes gathered together about two dozen organizations of very different kinds offering help to refugees, either French or foreign, lay or confessional, ranging from the American Friends Service Committee (Quakers) and the Unitarian Service Committee (Unitarians) to the French Red Cross and the Catholic Church of France, to seven Jewish organizations, and the CIMADE (*Comité Inter Mouvements Auprès des Évacués*), a member of the *Fédération Protestante* of France.

27 On Noel Field, see Subak, *Rescue and Flight*, xxv-xxvii, 84-86, *passim*.

28 Subak, *Rescue and Flight*, 85-87. The Marseille dispensary was located at 25 rue d’Italie. See American Unitarian Association, S*aving the Future in Europe:* [14].

29 Arrizabalaga, “La asistencia médica a los refugiados en Francia,” 72-86.

30 Ibid., 76-77.

31 Genizi, “Christian Charity,” 269-270; Arrizabalaga, “La asistencia médica a los refugiados en Francia,” 86-90.

32 Significantly, the USC devoted 2,500 dollars to the University of Minnesota Starvation Project, a clinical trial in which states of malnutrition then suffered by many people in Europe were experimentally reproduced in voluntary healthy individuals.

33 Genizi, “Christian Charity,” 271-273.

34 Wolfe, Barger, and Benison, *Cannon: Science and Society*, 516-517.

35 Faber, “Image Politics.”

36 Marcos Cueto, “An Asymmetrical Network: National and International Dimensions of the Development of Mexican Physiology,” *Journal of the History of Medicine and Allied Sciences* 71 (2016): 43-63.

37 One of those who made the publication of the Spanish version of Cannon’s *AAAS* (London: Kegan Paul and Co., 1932) possible was Rossend Carrasco i Formiguera, a pupil of his who revised it. *La sabiduría del cuerpo* (Mexico: Editorial Séneca, 1941) had been translated from English to Spanish by pharmacologist Jesús M. Bellido, mentioned above, from his exile at Toulouse. See Glick, “Walter B. Cannon”; Martínez Vidal and Sallent Del Colombo, “‘Making Science Understandable’”; Martínez Vidal and Sallent Del Colombo, “Ciencia en el exilio.”

38 Àlvar Martínez-Vidal and Antoni Adam-Donat, “*Spain in Exile*: l’Hospital Varsovia mis en scène,” in *L’Hôpital Varsovie*, 91-98.

39 Faber, “Image Politics.”

40 On the imprisonment of Barsky and other members of the JAFRC board, see ibid. On political repression against physicians during the Cold War Era, see Merlin Chowkwanyun, “‘The Neurosis That Has Possessed Us’: Political Repression in the Cold War Medical Profession,” *Journal of the History of Medicine and Allied Sciences* 73 (2018): 255-273.

41 “Nuestra protesta,” *Anales del Hospital Varsovia—Walter B. Cannon Memorial*, 1, 1948, 1 (our translation from Spanish).

42 The *Anales del Hospital Varsovia*, its quarterly hospital journal, only failed to include “Walter B. Cannon Memorial” in its title starting with its seventh issue (first term 1950). Arthur Schlesinger, Cannon’s son-in-law since 1940, claimed – without hiding his antipathy toward communists – that the name of Cannon had been instrumentalized “purely to raise funds in the United States.” Moreover, he stated that, “Socialist and trade union sources among Toulouse refugees [had] testified that the Varsovie Hospital was Communist-controlled and discriminated against non-Communists as both doctors and patients.” See Arthur Meier Schlesinger, *A Life in the Twentieth Century: Innocent Beginnings, 1917-1950* (Boston: Houghton Mifflin, 2000), 403.

43 Jordi Guixé Coromines, *La República perseguida. Exilio y represión en la Francia de Franco, 1937-1951* (Valencia: Publicacions de la Universitat de València, 2012), 403-405.

44 Phryné Pigenet, "L’opération ‘Boléro-Paprika’ ou la protection des étrangers à l’épreuve de la guerre froide," *Revue d’Histoire Contemporaine* 460 (1999): 296-310.

45 Guixé Coromines, *La República perseguida*, 420-433. Since 1970 its name has been Hôpital Joseph Ducuing; Olivier Munoz, “Théodolore Marie and Joseph Ducuing: deux approches de la lutte contre le cancer à Toulouse,” in *Lutter contre le cancer (1740-1960)*, ed. Didier Foucault (Toulouse: Éditions Privat, 2012), 283-311.

46 For a general view on this issue, see among others John Hedley Brooke, *Science and Religion: Some Historical Perspectives* (Cambridge: Cambridge University Press, 1991); John Hedley Brooke and Geoffrey Cantor, *Reconstructing Nature: The Engagement of Science and Religion* (Oxford: Oxford University Press, 1998); Paul Wood, ed., *Science and Dissent in England, 1688-1945* (Aldershot: Ashgate, 2004), particularly the chapters by Paul Wood (“Introduction: Stepping Out of the Merton’s Shadow,” 1-18) and John Hedley Brooke (“Science and Dissent: Some Historiographical Issues,” 19-37); José Montesinos and Sergio Toledo, ed., *Ciencia y religión en la Edad Moderna* (La Orotava: Fundación Canaria Orotava de Historia de la Ciencia, 2007). Specifically concerning the relationships between medicine and religion, see e.g., Ole Peter Grell and Andrew Cunningham, ed., *Medicine and the Reformation* (London: Routledge, 1993); Ole Peter Grell and Andrew Cunningham, ed., *Religio Medici: Medicine and Religion in Seventeenth Century* (London: Scholar Press, 1996); Ole Peter Grell and Andrew Cunningham, ed., *Medicine and Religion in Enlightenment Europe* (Aldershot: Ashgate, 2007).

47 Robert K. Merton, “Puritanism, Pietism and Science,” *Sociological Review* 28 (1936): 1-30; Robert K. Merton, “Science, Technology and Society in Seventeenth-Century England,” *Osiris* 4 (1938): 360-632; Charles Webster, *The Great Instauration: Science, Medicine and Reform, 1626-1660* (London: Duckworth, 1975).

48 On Unitarianism and the Unitarians, see “Unitarians,” *The Catholic Encyclopedia*, http://www.catholic.org/encyclopedia/view.php?id=11809; Catherine Robinson, “Face to Faith,” *The Guardian*, 28 March 2009, https://www.theguardian.com/commentisfree/2009/mar/28/unitarian-tim-berners-lee.

49 Brooke and Cantor, *Reconstructing Nature*, 247-281, quotation at 247.

50 John Hedley Brooke, “La ciencia en los unitarios,” in *Ciencia y religión en la Edad Moderna*, 253-271, quotation at 257.

51 Ibid., 258-259. For exemplary profiles of twentieth-century notable Unitarians, see Herbert F. Vetter, *Notable American Unitarians, 1936-1961* (Cambridge, MA: Harvard Square Library, 2007).

52 Cannon, *Way of an Investigator*, 16. On Huxley’s controversies with Gladstone and Wilberforce, see J. Vernon Jensen, *Thomas Henry Huxley: Communicating for Science* (Newark, NJ: Associated University Presses, 1991), 63-86, 111-125.

53 Cannon, *Way of an Investigator*, 16.

54 Ibid.

55 Saul Benison, A. Clifford Barger, and Elin L. Wolfe, *Walter B. Cannon: The Life and Times of a Young Scientist* (Cambridge, MA: Harvard University Press, 1987), 14-15.

56 Ibid., 26-29.

57 Cannon, *Way of an Investigator*, 17; Benison, Barger, and Wolfe, *Cannon: Life and Times of a Young Scientist*, 15. The physicist John Tyndall was a strong supporter of Darwin’s theory of evolution and of the separation of religion and science, as well as a close friend of Thomas H. Huxley. The philosopher Lewes actively participated in the Victorian discussion of Darwinism, positivism, and religious skepticism. Fiske was a US popularizer of Charles Darwin’s work.

58 For a general history of Harvard University, see Samuel Eliot Morison, *Three Centuries of Harvard, 1636-1936* (Cambridge, MA: Harvard University Press, 1936). On the crucial Unitarian influence on Harvard University from the early nineteenth century, see particularly 187-91, 242-245.

59 On the presidency of Charles W. Eliot, see Morison, *Three Centuries of Harvard*, 323-364. On his reform of Harvard Medical School, “one of Eliot’s earliest, most difficult and most important reforms” according to Morison, see ibid., 338-341, quotation at 338. The principle of academic intellectual freedom (*Lernfreiheit*), understood as the “duty of considering science and learning as an independent interest of the community,” had begun to be widely discussed at Harvard during the presidency of Josiah Quincy (1829-1845) under the stimulus of German exiles and students (see Morison, *Three Centuries of Harvard*, 254-259), and was revived again during the presidency of Abbot Lawrence Lowell (1909-1933) on the occasion of World War One (ibid., 454-456).

60 Stephen P. Shoemaker, “The Theological Roots of Charles W. Eliot’s Educational Reforms,” *Journal of Unitarian Universalist History* 31 (2006-2007): 30-45, particularly at 38-41.

61 Ibid., 41-42.

62 Eliot’s views are clearly shown in the speech “The Religion of the Future” he gave at the end of the 11^th^ session of the Harvard Summer School of Theology on 22 July 1909: “The religion of the future will not be based on authority, either spiritual or temporal.…There will be in the religion of the future no worship, express or implied, of dead ancestors, teachers, or rulers; no tribal, racial, or tutelary gods; no identification of any human being, however majestic in character, with the Eternal Deity.…In the religious life of the future the primary object will not be personal welfare or safety of the individual in this world or any other.…The religion of the future will not be propitiatory, sacrificial, or expiatory.…[It] will not perpetuate the Hebrew [*sic*] anthropomorphic representations of God….[It] will not be gloomy, ascetic, or maledictory.…this twentieth-century religion is not only to be in harmony with the great secular movements of modern society – democracy, individualism, social idealism, the zeal for education, the spirit of research, the modern tendency to welcome the new, the fresh powers of preventive medicine, and the recent advances in business and industrial ethics – but also in essential agreement with the direct personal teachings of Jesus as they are reported in the Gospels. The revelation He gave to mankind thus becomes more wonderful than ever.” See William Allan Neilson, ed., *Charles W. Eliot: The Man in his Beliefs* (New York: Harper and Brothers, 1926), II, 579-603, quotation at 602-603.

63 On the links between Cannon and Eliot, see Benison, Barger, and Wolfe, *Cannon: Life and Times of a Young Scientist*, 70, 82, 101, 113-114, 122-125, 129-130, *passim*.

64 Cannon, *Way of an Investigator*, 19.

65 Ibid., 17.

66 Benison, Barger, and Wolfe, *Cannon: Life and Times of a Young Scientist*, 50-73.

67 Cannon, *Way of an Investigator*, 21.

68 Benison, Barger, and Wolfe, *Cannon: Life and Times of a Young Scientist*, 2.

69 Cannon introduced the concept of homeostasis in 1929. On the similarities and differences between this concept and Claude Bernard’s *milieu intérieur*, see Mathieu Arminjon, “Birth of the Allostatic Model: From Cannon’s Biocracy to Critical Physiology,” *Journal of the History of Biology* 49 (2016): 393-423.

70 Cannon, *Way of an Investigator*, 154-159. For the context of the antivivisectionist agitation and of Cannon’s defense of medical research, see Benison, Barger, and Wolfe, *Cannon: Life and Times of a Young Scientist*, 168-186, 187-201. Cannon's papers concerning this issue are preserved in the American Philosophical Association Library (Mss.B.C163.1).

71 Benison, Barger, and Wolfe, *Cannon: Life and Times of a Young Scientist*, 151-152.

72 See Adrian Desmond and James Moore, *Darwin’s Sacred Cause: How a Hatred of Slavery Shaped Darwin’s Views on Human Evolution* (New York: Houghton Mifflin Harcourt, 2009).

73 Maria I. Diedrich, *Cornelia James Cannon and the Future American Race* (Amherst: University of Massachusetts Press, 2010), 30, 33, 52, 106, *passim*.

74 Wolfe, Barger, and Benison, *Cannon: Science and Society*, 134-139, 441.

75 Raymond J. Cunningham, “The Emmanuel Movement: A Variety of American Religious Experience,” *American Quarterly* 14 (1962): 48-63.

76 Ida Maud Cannon, *Social Work in Hospitals: A Contribution to Progressive Medicine* (New York: Russell Sage Foundation, 1917); Amy Dahlberg Chu, “Ida Maud Cannon,” *Dictionary of Unitarian and Universalist Biography*, Unitarian Universalist History and Heritage Society (UUHHS) 1999-2020: https://uudb.org/articles/idamaudcannon.html.

77 See Marian Cannon Schlesinger, *Snatched from Oblivion: A Cambridge Memoir* (Boston: Little, Brown and Co., 1979), 109.

78 Cannon, *Way of an Investigator*, 164.

79 Ibid., 164-165. For a thorough analysis on Cannon’s views about the relationship between the biological homeostasis and the social one, see Mathieu Arminjon, “Introduction: Homéostasie, stress et société: Walter B. Cannon aux fondements des déterminants sociaux de la santé,” in *Walter B. Cannon, Conférences sur les émotions et l’homéostasie, Paris, 1930* (Lausanne: Éditions BHMS, 2020), particularly at 89-97.

80 Wolfe, Barger, and Benison, *Cannon: Science and Society*, 261-262, 570 n. 30; Arminjon, “Introduction,” 77-81.

81 Walter B. Cannon, “The Body Physiologic and the Body Politic,” *Science* 93: 2401 (3 January 1941): 1-10. The AAAS had been founded in 1848 to promote scientific dialogue, collaboration, and scientific progress. By the time of Cannon’s speech, the AAAS had become the largest and most venerable scientific organization in the US, and was made up of 19,000 members and some 750,000 affiliates from all scientific disciplines. See Peter J. Kuznick, *Beyond the Laboratory: Scientists as Political Activists in 1930s America* (Chicago: University of Chicago Press, 1987), 44, 152, 320; Wolfe, Barger, and Benison, *Cannon: Science and Society*, 440-4; Arminjon, “Birth of the Allostatic Model,” 405-409.

82 Cannon, *Way of an Investigator*, 165, 175. For the Wicht Club and Cannon’s friendships in Cambridge, MA, see Benison, Barger, and Wolfe, *Cannon: Life and Times of a Young Scientist*, 135-150.

83 Arminjon, “Birth of the Allostatic Model,” 406-407.

84 On the AAAS and the Science and Society movement, see Kuznick, *Beyond the Laboratory*, 71-105. On Boas and the mobilization of North American scientists against fascism, see ibid., 171-194, 324-332. See also Peter J. Kuznick, “The Birth of Scientific Activism,” *Bulletin of the Atomic Scientists* 44 (1988): 39-43; Jaume Sastre-Juan, “Philanthropy, Mass Media and Cultural Hegemony: The Rockefeller Foundation and the Politics of Science Popularization in the 1930s,” in *Cultural Hegemony in a Scientific World. Gramscian Concepts for the History of Science*, ed. Massimiliano Badino and Pietro Daniele Omodeo (Leiden: Brill, 2021).

85 Kuznick, *Beyond the Laboratory*, 101-102, 184-185, 190-194, 197, 220-221, 224-225, 235, 238, 243-247, 258-260, 305, 342-343; Wolfe, Barger, and Benison, *Cannon: Science and Society*, 440-441, 450-452, 601-602.

86 See its transcription in Wolfe, Barger, and Benison, *Cannon: Science and Society*, 602.

87 Ibid., 492-494, 498-500. On the American-Soviet Medical Society, see Walter J. Lear, “Hot War Creation, Cold War Casualty: The American-Soviet Medical Society, 1943-1948,” in *Making Medical History: The Life and Times of Henry E. Sigerist*, ed. Elisabeth Fee and Theodore M. Brown (Baltimore: Johns Hopkins University Press, 1997), 259-287. On Cannon’s involvement in this society, see ibid., 263-266, 271, 273.

88 Henry Sigerist, “‘Walter B. Cannon’ (Memorial meeting October 24, 1945, New York Academy of Medicine),” *American Review of Soviet Medicine* 3 (1945): 282-288, quotation at 282; Kuznick, *Beyond the Laboratory*, 150-157, 160-170; Wolfe, Barger, and Benison, *Cannon: Science and Society*, 498-500.

89 Cannon, *Way of an Investigator*, 163-164; Arminjon, “Birth of the Allostatic Model,” 410-412.

90 Kuznick, *Beyond the Laboratory*, 62-63; Kuznick, “The Birth of Scientific Activism,” 40.

91 Schlesinger, *A Life in the Twentieth Century*, 183.

92 On US scientists’ disappointment in the New Deal in the 1930s, see Kuznick, *Beyond the Laboratory*, 63-64, 256-257; Kuznick, “Birth of Scientific Activism,” 40.

93 On the counter-hegemonic solidarity pattern, see Anne-Emanuelle Birn and Theodore M. Brown, “Across the Generations: Lessons from Health Internationalism,” in *Comrades in Health*, 311-312.

94 Gary Werskey, *The Visible College: A Collective Biography of British Scientists and Socialists of the 1930s* (London: Allen and Unwin, 1978); Kuznick, *Beyond the Laboratory*.

95 Saul Benison, “Walter B. Cannon and the Politics of Medical Science, 1920-1940,” *Bulletin of the History of Medicine* 65 (1991): 234-251, quotation at 250-251.

96 Kuznik, *Beyond the Laboratory*; Stephen J. Cross and William R. Albury, “Walter Cannon, I. J. Henderson, and the Organic Analogy,” *Osiris* 3 (1987): 165-192.

97 Arminjon, “Birth of the Allostatic Model,” 405-413, quotation at 409.

98 Misleading readings of Cannon’s biocracy by historians have been documented. The one by Georges Canguilhem is discussed in Arminjon, “Introduction,” 89-97. The one by Stephen J. Cross and William R. Albury is discussed in Christopher Lawrence and George Weisz, “Medical Holism: The Context,” in *Greater Than the Parts: Holism in Biomedicine, 1920-1950*, ed. Christopher Lawrence and George Weisz (Oxford: Oxford University Press, 1998), 1-22; Arminjon, “Birth of the Allostatic Model,” 409-412.

99 Benison, Barger, and Wolfe, *Cannon: Life and Times of a Young Scientist*; Wolf, Barger, and Benison, *Cannon: Science and Society*.

100 Wolfe, Barger, and Benison, *Cannon: Science and Society*, 457-466.

101 Ibid., 517.

102 Cannon, *Way of an Investigator*, 9.

103 On the difficulties resulting from the use of the autobiographies as a source for the history of life sciences, see Rena Selya, “Primary Suspects: Reflections on Autobiography and Life Stories in the History of Molecular Biology,” in *The History and Poetics of Scientific Biography*, ed. Thomas Söderqvist (London: Routledge, 2007), 199-206.

104 The First Parish in Cambridge. The First Church (Unitarian), “Dr. Cannon Memorial Hospital Fund,” *Newsletter*, 16 January 1947, folder 712, box 54, Cannon’s papers, CML.

105 Chu, “Ida Maud Cannon.”

106 “We have received the following letter from Dr. Frederick May Eliot, Co-Chairman of the Dr. Walter B. Cannon Memorial Hospital Fund: ‘150,000 Spanish refugees from Franco fascism are homeless and half starved. Many of them are in dangerous physical condition – unable to get help from the Red Cross, UNRRA, or government authorities. These helpless people are entirely dependent on private help from people like ourselves. Now, to help bring desperately needed medical care to these valiant fighters for democracy, the Boston Chapter of the Joint Antifascist Refugee Committee has organized a Dr. Walter B. Cannon Memorial Hospital Fund. The money raised (which will be administered by the Unitarian Service Committee in France) will alleviate the sufferings of these brave people, and will also establish a fitting memorial to a great scientist and humanitarian – Dr. Walter B. Cannon, who was an active friend of the Spanish Republicans from the very beginning. Contributions may be sent to the Dr. Walter B. Cannon Memorial Hospital Fund, room 201, 14 Beacon Street, Boston 8. Dr. Cannon was a member of our congregation and a pew holder for many years.’” See The First Parish in Cambridge. The First Church (Unitarian), “Dr. Cannon Memorial Hospital Fund.”

107 The Unitarian Church minister Frederick May Eliot would be the president of the American Unitarian Association for twenty years (1938-1958). He happened to be related to Charles William Eliot whose previously mentioned forty-year long presidency of Harvard University (1869-1909) is crucial to understanding the critical influence of a singularly liberal Unitarian culture on this university. On Frederick May Eliot, see Subak, *Rescue and Flight*, 13, *passim*; Eliot's entry in the *Dictionary of Unitarian and Universalist Biography*: http://uudb.org/articles/frederickmayeliot.html.

## Notes

Email: alvar.martinez@uv.es

